# Supplementary material for: Explicitly Correlated Double-Hybrid DFT: A Comprehensive Analysis of the Basis Set Convergence on the GMTKN55 Database
Source: J Chem Theory Comput. 2022 Sep 13;18(10):5978–91. doi: 10.1021/acs.jctc.2c00426 (PMC9558368; doi:10.1021/acs.jctc.2c00426)
Supplement: Supplementary file 1 — ct2c00426_si_001.pdf [file ct2c00426_si_001.pdf]

Supporting Information: Explicitly correlated double hybrid DFT: a comprehensive analysis of the basis set convergence on the GMTKN55 database

Nisha Mehta and Jan M. L. Martin*

Department of Molecular Chemistry and Materials Science, Weizmann Institute of Science, Reḥovot, Israel

E-mail: gershom@weizmann.ac.il

Contents

SI.1 How to run DHDF-F12 calculations **S-3**
SI.1.1 Sample input for B2GP-PLYP-F12: S-3
SI.1.2 Sample input for revDSD-PBEP86-F12: S-3

SI.2 Analysis of weighted total mean signed deviation (WTMSD2) **S-4**

SI.3 Basis set convergence of B2GP-PLYP PT2 correlation component of Ne atom **S-6**

References **S-7**

SI.1 How to run DHDF-F12 calculations

SI.1.1 Sample input for B2GP-PLYP-F12:

```
gthresh,energy=1d-9, throvl=1d-9
ANGSTROM
geomtyp=xyz
geom={
include struc.xyz
}
! end geometry
thecharge=0
multiplicity=1

basis,avdz-f12
{DF-KS,b,lyp;dh,0.65,0.36;wf,spin=$multiplicity-1,CHARGE=$thecharge;}
eksf12=ENERGY;
DF-mp2-f12, GEM_BETA=0.9
! For close shell systems
if($multiplicity.eq.1)then
eglptf12=(ENERGY-ENERGR)*0.36
ecabs=EF12_SINGLES
else
! For close shell systems
eglptf12=(ENERG_VV+ENERG_CC+ENERG_CV)*0.36
ecabs=EF12_SINGLES
endif
ETOT=eksf12+eglptf12+ecabs
show,*
!Note that the DFT-D3(BJ) correction is not included in this molpro input.
```

SI.1.2 Sample input for revDSD-PBEP86-F12:

```
gthresh,energy=1d-9
ANGSTROM
geomtyp=xyz
geom={
include struc.xyz
}
! end geometry
thecharge=0
multiplicity=1

cDFTc=0.4210
cXHF=0.69
c2ab=0.5922
c2ss=0.0636

basis,vdz-f12
{DF-ks,pbex,p86;dh,cXHF,1.00-cDFTc; wf,spin=$multiplicity-1,CHARGE=$thecharge;}
EKS=ENERGY;
DF-mp2-f12,scsfacs=c2ab/(1.00-cDFTc),scsfact=c2ss/(1.00-cDFTc),  GEM_BETA=0.9
SCSMP2=(EMP2_SING+EMP2_TRIP/3)*1.02280+EMP2_TRIP*2/3*0.10984
SCSF12=(EF12_SING+EF12_TRIP/3)*1.02280+EF12_TRIP*2/3*0.10984
ECABS=EF12_SINGLES
ETOT=EKS+ECABS+(SCSMP2+SCSF12)*0.5790
show, *
! This is the end of Molpro input
! Note that the DFT-D4 dispersion correction is not included in this molpro input.
```

SI.2 Analysis of weighted total mean signed deviation (WTMSD2)

Table S1: WTMSD2 values (kcal/mol) of conventional and explicitly correlated B2GP-PLYP-D3(BJ) for GMTKN55 and its categories relative to Ref.[S1] reference data. A positive sign indicates overestimation.

	B2GP-PLYP-D3(BJ)					
	WTMSD2	THERMO	BARRIERS	LARGE	CONF	INTERMOL
VDZ	2.738	-0.862	-0.536	-0.331	2.343	2.124
VDZ*	3.453	-0.134	-0.396	-0.331	2.343	1.970
VDZ^m	1.083	-0.134	-0.396	-0.319	0.527	1.405
VTZ	1.382	-0.279	-0.289	-0.040	0.864	1.126
VTZ*	1.759	0.114	-0.195	-0.040	0.864	1.015
VTZ^m	0.910	0.114	-0.195	-0.048	0.325	0.714
VQZ	0.926	-0.084	-0.223	0.010	0.448	0.774
VQZ*	1.180	0.138	-0.146	0.010	0.448	0.729
VQZ^m	0.860	0.138	-0.146	0.009	0.289	0.570
V{T,Q}Z	1.158	0.007	-0.242	0.033	0.367	0.994
V{T,Q}Z*	1.340	0.181	-0.145	0.033	0.367	0.905
V{T,Q}Z^m	1.037	0.181	-0.145	0.032	0.270	0.700
V5Z*	0.745	0.154	-0.123	0.006	0.290	0.418
V5Z^m	0.688	0.154	-0.123	0.006	0.265	0.387
V{Q,5}Z*	0.735	0.183	-0.118	0.004	0.235	0.431
V{Q,5}Z^m	0.743	0.183	-0.118	0.004	0.262	0.413
def2-TZVPP	0.682	-0.184	-0.202	-0.004	0.512	0.560
def2-TZVPP*	0.978	-0.007	-0.127	-0.004	0.512	0.604
def2-TZVPP^m	0.730	-0.007	-0.127	-0.009	0.290	0.583
def2-TZVPPD	0.844	-0.040	-0.130	0.044	0.274	0.696
def2-QZVPP	0.452	0.004	-0.148	0.012	0.312	0.272
def2-QZVPP*	0.644	0.079	-0.108	0.012	0.312	0.349
def2-QZVPP^m	0.611	0.079	-0.108	0.012	0.284	0.344
def2-QZVPPD	0.655	0.076	-0.109	0.023	0.270	0.394
def2-{T,Q}ZVPP	0.634	0.094	-0.157	0.022	0.277	0.398
def2-{T,Q}ZVPP*	0.739	0.136	-0.112	0.022	0.277	0.416
def2-{T,Q}ZVPP^m	0.682	0.136	-0.112	0.023	0.298	0.336
def2-{T,Q}ZVPPD	0.565	0.141	-0.107	0.007	0.291	0.233
VDZ-F12	5.883	0.880	0.323	0.916	1.539	2.225

	B2GP-PLYP-F12-D3(BJ)					
	WTMSD2	THERMO	BARRIERS	LARGE	CONF	INTERMOL
AVDZ-F12	0.737	0.145	-0.127	-0.009	0.260	0.468
VDZ-F12	0.741	0.141	-0.125	-0.003	0.268	0.459
VDZ-F12*	0.753	0.155	-0.127	-0.003	0.268	0.460
VTZ-F12	0.734	0.168	-0.128	0.006	0.279	0.409
VTZ-F12*	0.755	0.177	-0.129	0.006	0.279	0.422
V{D,T}Z-F12	0.748	0.177	-0.133	0.008	0.283	0.413
V{D,T}Z-F12*	0.774	0.184	-0.133	0.008	0.283	0.431
VQZ-F12	0.682	0.175	-0.123	0.005	0.261	0.365
VQZ-F12*	0.689	0.180	-0.120	0.005	0.260	0.364
V{T,Q}Z-F12	0.683	0.177	-0.123	0.004	0.259	0.365
V{T,Q}Z-F12*	0.685	0.181	-0.119	0.004	0.259	0.360

Table S2: WTMSD2 values (kcal/mol) of conventional and explicitly correlated B2GP-PLYP-D3(BJ) for GMTKN55 and its categories relative to the B2GP-PLYP-F12-D3(BJ)/V{T,Q}Z-F12* reference data. A positive sign indicates overestimation.

	B2GP-PLYP-D3(BJ)					
	WTMSD2	THERMO	BARRIERS	LARGE	CONF	INTERMOL
VDZ	1.949	-1.040	-0.429	-0.350	2.055	1.712
VDZ*	2.693	-0.310	-0.285	-0.350	2.055	1.584
VDZ^m	0.345	-0.310	-0.285	-0.339	0.264	1.015
VTZ	0.701	-0.458	-0.175	-0.046	0.600	0.781
VTZ*	1.077	-0.064	-0.078	-0.046	0.600	0.666
VTZ^m	0.235	-0.064	-0.078	-0.053	0.067	0.364
VQZ	0.246	-0.261	-0.106	0.007	0.188	0.417
VQZ*	0.501	-0.038	-0.028	0.007	0.188	0.372
VQZ^m	0.182	-0.038	-0.028	0.005	0.031	0.213
V{T,Q}Z	0.464	-0.170	-0.127	0.030	0.107	0.623
V{T,Q}Z*	0.638	0.005	-0.028	0.030	0.107	0.523
V{T,Q}Z^m	0.337	0.005	-0.028	0.030	0.011	0.318
V5Z*	0.076	-0.022	-0.004	0.002	0.032	0.068
V5Z^m	0.019	-0.022	-0.004	0.001	0.007	0.036
V{Q,5}Z*	0.042	0.001	0.001	-0.001	-0.023	0.063
V{Q,5}Z^m	0.048	0.001	0.001	-0.001	0.002	0.044
def2-TZVPP	0.009	-0.358	-0.085	-0.008	0.250	0.211
def2-TZVPP*	0.314	-0.181	-0.008	-0.008	0.250	0.262
def2-TZVPP^m	0.074	-0.181	-0.008	-0.013	0.034	0.242
def2-TZVPPD	0.183	-0.212	-0.012	0.043	0.019	0.345
def2-QZVPP	-0.228	-0.173	-0.030	0.009	0.054	-0.089
def2-QZVPP*	-0.024	-0.097	0.011	0.009	0.054	-0.001
def2-QZVPP^m	-0.060	-0.097	0.011	0.008	0.024	-0.006
def2-QZVPPD	-0.016	-0.100	0.011	0.021	0.011	0.042
def2-{T,Q}ZVPP	-0.060	-0.084	-0.039	0.019	0.017	0.026
def2-{T,Q}ZVPP*	0.049	-0.042	0.007	0.019	0.017	0.047
def2-{T,Q}ZVPP^m	-0.008	-0.042	0.007	0.020	0.039	-0.032
def2-{T,Q}ZVPPD	-0.110	-0.036	0.012	0.003	0.032	-0.122

	B2GP-PLYP-F12-D3(BJ)					
	WTMSD2	THERMO	BARRIERS	LARGE	CONF	INTERMOL
AVDZ-F12	0.059	-0.035	-0.008	-0.014	0.001	0.114
VDZ-F12	0.069	-0.039	-0.005	-0.007	0.010	0.111
VDZ-F12*	0.081	-0.026	-0.007	-0.007	0.010	0.112
VTZ-F12	0.048	-0.013	-0.009	0.002	0.019	0.050
VTZ-F12*	0.071	-0.005	-0.010	0.002	0.019	0.065
V{D,T}Z-F12	0.059	-0.005	-0.014	0.005	0.024	0.050
V{D,T}Z-F12*	0.087	0.002	-0.014	0.005	0.024	0.070
VQZ-F12	0.001	-0.007	-0.003	0.000	0.002	0.009
VQZ-F12*	0.006	-0.002	0.000	0.000	0.002	0.006
V{T,Q}Z-F12	0.000	-0.005	-0.003	0.000	0.000	0.008
V{T,Q}Z-F12*	REF	REF	REF	REF	REF	REF

SI.3 Basis set convergence of B2GP-PLYP PT2 correlation component of Ne atom

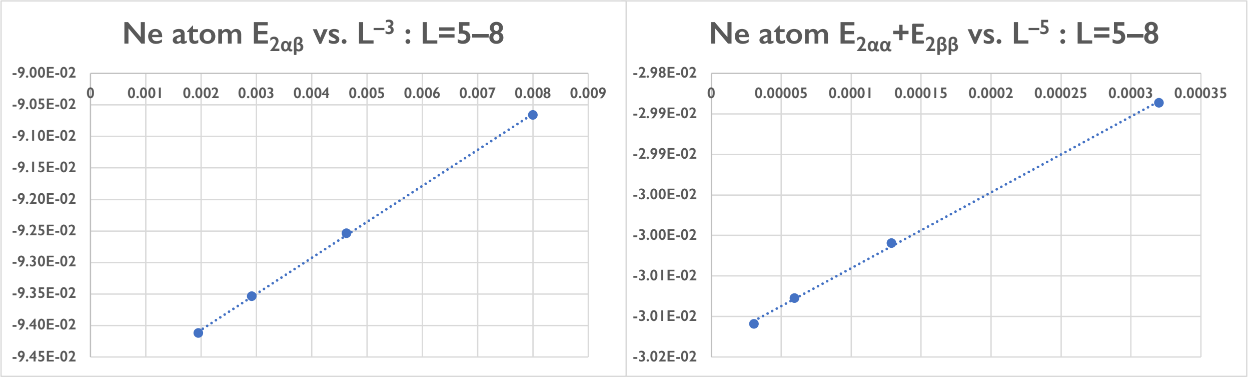

Figure S1: B2GP-PLYP same-spin and opposite-spin PT2 components for neon atom with Petersson's nZaP basis sets as a functions of L=n

References

(S1) Goerigk, L.; Hansen, A.; Bauer, C.; Ehrlich, S.; Najibi, A.; Grimme, S. *Phys. Chem. Chem. Phys.* **2017**, *19*, 32184–32215.
